# Determination of CRP blood level in type 1 diabetic patients and the effect of aerobic and resistance training on the level of this biomarker

**DOI:** 10.22088/cjim.13.1.38

**Published:** 2022

**Authors:** Yousef Moghaddasi Kouchaksaraei, Farshad Ghazalian, Saeid Abediankenari, Khosro Ebrahim, Hossein Abednatanzi

**Affiliations:** 1 Department of Sports Physiology, Science and Research Branch, Islamic Azad University, Tehran, Iran; 2 Department of Physical Education and Sports Sciences, Science and Research Branch, Islamic Azad University, Tehran, Iran; 3 Immunogenetics Research Center, Mazandaran University of Medical Sciences, Sari, Iran; 4 Department of Exercise Physiology, Shahid Beheshti University, Tehran, Iran

**Keywords:** Type 1 diabetes, CRP protein, Aerobic and resistance training

## Abstract

**Background:**

Increasing prevalence of diabetes and its complications, including cardiovascular problems, increase the cost of health care. With proper planning to change lifestyle, like costs and complications of type 1 diabetes could be diminished. The present study investigated the effect of aerobic and resistance training on blood CRP level of type 1 diabetic patients as a protective marker on cardiovascular cells.

**Methods:**

In this descriptive cross-sectional study, 32 patients with type 1 diabetes were divided into two groups of aerobic and resistance exercise training. Serum CRP levels were measured in all patients before and after exercise. Data were analyzed using Mann-Whitney, Bootstrap and SPSS tests.

**Results:**

In this study, for abnormal data, Bootstrap method was used, which created an acceptable confidence interval. And using analysis of variance to control the effect of CRP (interfering) level before and after exercise was not significant (P=0.37).

**Conclusion:**

Considering the relationship between exercise training with CRP level in type 1 diabetic patients specially in aerobic training group as well as CRP level according to the training program condition, it can be concluded that there is not effective relationship between this biomarker and exercise training in type 1 diabetic patients.

Type 1 or insulin-dependent diabetes is an endocrine disease of childhood and adolescence (1). The prevalence of type 1 diabetes is increasing in all parts of the world, the main cause of which is unknown (2). In Iran, diabetes is at the top of non-communicable diseases in the country (3, 4). Adolescents often struggle with changes in blood sugar due to developmental conditions of puberty and reduced adherence to treatment due to psychological conditions during puberty at this age (5, 6). Diabetes is known as a debilitating disease due to its many consequences and various complications among the people. Therefore, more attention should be paid to diabetes-related health care spending reduction programs (8, 7). Exercise has a significant effect on food metabolism, especially in lowering blood sugar levels, which is an important therapeutic value for diabetics (9). Type 1 diabetes is caused by a lack of insulin due to the autoimmune destruction of pancreatic beta cells. In these patients, a lack of secretion or decreased insulin function leads to impaired metabolism of carbohydrates, fats and proteins. The annual incidence of type 1 diabetes in Iran is estimated at 7.3 cases per 100,000 people. This figure varies from 1 to 35 cases per 100,000 population under the age of 14 worldwide (10). There is much controversy about the effect of exercise on type 1 diabetics.

The existence of these contradictions, on the one hand, and the lack of familiarity with how to prescribe exercise to these patients, on the other hand, deprives type 1 diabetics of the benefits of participating in sports activities. Studies show that patients with uncomplicated type 1 diabetes with good metabolic control can participate in all levels of exercise (12, 11). Regular exercise increases the rate of metabolism and the rate of glucose transport and transport in the body and improves cell sensitivities to injected insulin (9, 13). Also, regular exercise reduces the risk of coronary heart disease by affecting blood lipids. The most common cause of death in people with diabetes is coronary heart disease. Research shows that regular exercise reduces this risk by up to 50%. For this reason, the American Diabetes Association (ADA) has identified lack of regular exercise has been identified as a fourth risk factor for coronary heart disease, and the other three risk factors are high blood pressure, smoking and high cholesterol (14). 

C-reactive protein (CRP) is an acute-phase reactant and is elevated in inflammatory states. A previous study has suggested that CRP levels are elevated in type 1 diabetes (15). In another study (In 2020) children with type 1 diabetes have elevated high-sensitivity C-reactive protein compared with a control group (16). In another study, plasma concentrations of C-reactive protein were higher in type I diabetic patients without (clinical) macroangiopathy than in control subjects, probably due to a chronic hepatic inflammatory response. The correlation of C-reactive protein with markers of endothelial dysfunction suggests a relation between activation of the endothelium and chronic inflammation (17). CRP protein is an acute phase reactive protein that is made during inflammatory processes. Systemic activation of the inflammatory process is the body's appropriate response to trauma or disease. CRP typically increases within hours of the onset of infection and / or inflammation. This protein is considered as part of the acute phase response, which is activated exclusively by the disease. This protein is produced as part of the liver phase response by various cytokines, including interleukin 1 beta (IL1-β), tumor necrosis factor alpha (TNF α), and interleukin IL6 .These inflammatory cytokines are produced by different cells, but the most important are macrophages and monocytes at the site of inflammation; The appearance, increase or decrease in the amount of each of the acute phase proteins during a disease is different and independent of each other. CRP is commonly used to identify and monitor the progression of inflammatory processes due to infection (18, 19). On the other hand, CRP levels can be used as a marker for severely non-infectious patients; For example, research by Michel et al. Showed that increased CRP levels in patients with esophageal cancer were associated with tumor progression (20), and in other studies, a diagnostic role in gastrointestinal cancers (21) or ovaries were shown (15). Wullstein et al. used CRP to monitor graft rejection (22). Werner et al. also showed that the severity of pancreatitis is associated with increased CRP levels (23).

Type 1 diabetes is thought to be an acute phase disease in which the concentration of cytokines produced by macrophages, adipose tissue and endothelium is increased by stimuli such as overeating - hyperglycemia. Cytokines, especially TNFα, IL-1, IL-IL-6, affect the liver, increasing VLDL and decreasing HDL, and stimulating the release of acute phase proteins such as fibrinogen and CRP, which have an atherosclerotic effect. The effect of cytokines on adipose tissue is to release leptin and, on the brain, to release ACTH and then cortisol. ACTH and cortisol are involved in obesity, blood pressure and insulin resistance. Prolonged secretion of cytokines interferes with the secretion of insulin from pancreatic beta cells. Serum concentrations of acute phase proteins such as sialic acid, glycoprotein A-1, CRP and amyloid A, and cortisol have been shown to be high in diabetes. Concentrations are minimal in non-diabetics, moderate in diabetics without metabolic syndrome, and maximal in diabetics with metabolic syndrome. Urinary secretion of albumin is of the same grade, and microalbuminuria is thought to be a component of the acute phase response. It is important in diabetes. Because it transfers apolipoprotein A1 from HDL 3, and binds HDL to macrophages, it acts as a signal that transports HDL from the liver to macrophages for tissue repair. Increased catabolism can reduce HDL uptake in diabetes. Macrophage in the formation of atherosclerotic plaques can be the cause of vascular disease in diabetes. Other acute phase proteins that are elevated in diabetes include fibrogen (a potent risk factor for cardiovascular disease), von Willebrand factor, and Camplan components, PAL-1. There is evidence that LP (a) lipoprotein, which is associated with cardiovascular disease in diabetes, is an acute phase protein (24).

Due to the contradictory results of different studies and the prevalence of this disease in our country, the present study can be very important for type 1 diabetic patients and diabetes-related centers to better control the disease. Also, considering the role of CRP as a prognostic marker, we sought to demonstrate the association of this biomarker with exercise in patients with type 1 diabetes. It should be noted that the present study investigated the effect of a course of aerobic and resistance training on the CRP blood level of type 1 diabetic patients referred to medical clinics in Mazandaran province.

## Methods

**Type of research:** This study, with the ethical code IR.MAZUMS.REC.1398.6489 was a cross-sectional descriptive clinical trial in which the pre-test, post-test design was used. Statistical sample was performed among type 1 diabetic patients under the supervision of Mazandaran University of Medical Sciences.

**Method of conducting research:** This study has three parts. In order: selecting people and obtaining informed consent and visits, holding explanatory sessions of subjects and physical analysis and division into two groups (aerobic and resistance) and preparing a sample of zero pre-test (5 ml of peripheral blood) to the title of the control group of all patients in both groups is program training and training steps and finally post-test sampling and laboratory analysis of samples and statistical analysis (25). In this study, we took peripheral blood from these patients before (assumption zero or control group) as well as after exercising, and using the kit, we determined the CRP level and also compared that doing exercises is different between the two training groups? And does exercise affect the blood level of this biomarker?

In this study, because our subjects were young, they were less likely to develop infection and inflammation, and as a result, the CRP level of many of these individuals was zero, and a small number (three) of the subjects in this study had significant CRP. They were in their blood sample, which decreased after regular exercise. And other studies have shown that exercise can reduce CRP levels in people with infections or inflammation. The condition for the continued presence of the subjects (patients) in this plan is that they do not have acute infectious disease and in case of acute infectious disease, they are excluded from the study. Statistical population, sampling method and number of samples. The statistical population that was eligible to participate in the study was purposefully selected. For this purpose, first age-type type 1 diabetic patients are invited and after a face-to-face interview and full explanation of the study stages, their recent quarterly tests are reviewed and these people are in Sari Bu Ali Sina Hospital. They had a medical record for more than 2 years and are under the supervision of a specialist doctor. They were invited to cooperate. The number of samples was 32 randomly in the aerobic group of 15 people and the resistance group of 17 people and the age range of patients was 10 to 25 years and there was no significant difference in the mean age between the two groups.

And the sample size formula is displayed below.

$n_{1}=n_{2}=\frac{2\times {\left(Z_{\frac{a}{2}}+Z_{\beta }\right)}^{2}\times \sigma^{2}}{{\left(d_{1}d_{2}\right)}^{2}}=18\mathrm{.a}=0.05\mathrm{. \beta}=0.2\mathrm{.\sigma}=0.00125.d_{1}=0.001.d_{2}=0.0022$

Before receiving the consent of the subjects, the necessary information about the nature, manner of conducting the research, possible risks and the points that must be observed to participate in this study were provided to them orally and in writing. The Ethics Committee of Mazandaran University of Medical Sciences approved this study.

**Table 1:** Demographic and descriptive table in terms of gender between the two groups

	**EXercisetype**	
	**Aerobic**	**resistance**
**Gender **n (%) Male Female	8(34.8) 7(77.8)	15(65.2) 2(22.2)
Total n (%)	15(46.9)	17(53.1)

**Figure 3:**
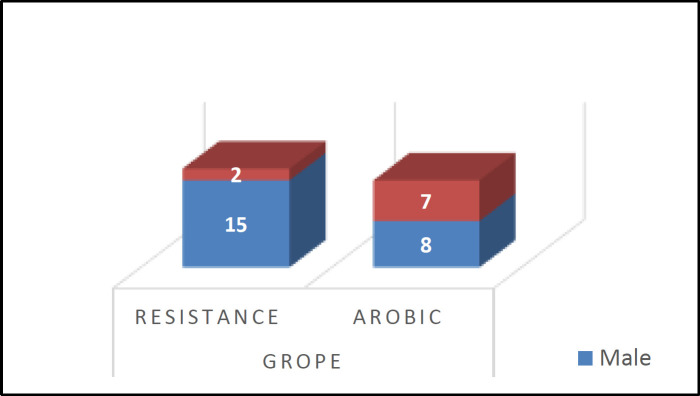
Specification image of CRP measuring kit

**Aerobic exercise protocol** **:** The aerobic exercise program includes running outdoors for 8 weeks for 3 sessions per week, starting at 30 minutes with an intensity of 60% of heart rate and ending with 60 minutes with an intensity of 85% of maximum heart rate in the eighth week. In each training session, approximately 10 minutes of warm-up (jogging: 2-4 minutes, warm-up of the joints: 2 minutes and performing stretching exercises: 4 minutes) and 5 minutes of cooling time was considered. Maximum heart rate using the formula: [Age-220] was obtained and during the performance, the intensity of training was given to this group in the form of a table according to the percentage of maximum heart rate written in the training protocol.

**Strength training program** **:** The strength training program consisted of 8 weeks for 3 sessions per week. The intensity of training was determined based on the percentage of a maximum repetition (1RM). The training started with 1 set of 15 repetitions and 60% of a maximum repetition and increased to 85%. In 2 sets, 12 repetitions in the eighth week ended. Due to the fact that the subjects participating in this study are non-athletes, a maximum replication test (1RM) was used to prevent possible injuries. The exercises are performed in 8 stations and were performed in the form of upper body, lower body and combination exercises during this protocol. This program includes movements: car abdomen, car chest press, car shelf, front slash, car front arm, car shoulder press, car ankle, car back foot, car foot press, car leg.

**Check serum CRP levels:** According to the Bionik kit shown in figures (3-2). 

**Methods and tools of data analysis:** To analyze the data, first using descriptive methods including mean standard deviation for quantitative data with normal and middle distributions and the first and third quarters for abnormal quantitative variables and many tables for qualitative variables are summarized. Then, to compare the groups, qualitative variables should be compared using chi-square tests. Mann-Whitney and Bootstrap tests were used to normalize the data. To perform this analysis, SPSS 23 software was used at a significance level of 0.05.

## Results

**Serum CRP levels:** For abnormal data, the Bootstrap method was used, which creates an acceptable confidence interval. And using analysis of variance to control the effect of CRP (interfering) level before and after exercise was not significant (P=0.37).

**Table 4:** CRP levels between aerobic and resistance groups before and after exercise

**p**	**Mean** **±** **standard deviation**	**Group**	
0.88	4.02±1.58	Aerobic	Before
	3.26±1.3	Resistance	
0.91	2.57±0.68	Aerobic	after
	1.99±0.12	Resistance	
0.37	-1.44±0.99	Aerobic	Difference
	-1.28±1.27	Resistance	

## Discussion

Given the growing prevalence of type 1 diabetes worldwide and the fact that diabetes is one of the diseases in which the immune system is activated; the theory is now reinforced that one of the main factors linking cardiovascular disease and diabetes is the activation of the immune system and inflammation. In type 1 diabetics, an increase in CRP markers can also be detected. Reactive C protein (CRP) increases in acute inflammatory conditions such as infectious diseases and connective tissue diseases. The present study investigated the effect of a course of aerobic and resistance training on CRP blood level in type 1 diabetic patients that referred to medical clinics in Mazandaran province.

In this study, we found that using abnormal data, the Bootstrap method was used, which created an acceptable confidence interval. Using analysis of variance to control the effect of CRP (interfering) level before and after exercise was not significant (P=0.37). Due to significant advances in biomarker exploration, only a small number of biomarkers have been found to be widely used in clinical practice such as troponin T, troponin I, Nt-proBNP and natriuretic peptide type B (BNP) (26).

It has been shown that various factors can affect the secretion of adipokines, including exercise. Exercise can also be effective in different ways depending on its intensity and duration (27). Exercise-induced weight loss results in increased levels of anti-inflammatory adipocytokines, decreased CRP levels, and improved insulin sensitivity (28). Exercise reduces CRP status. In a study by Lakka et al. (2005), (29). They observed a decrease in CRP levels in inactive individuals after a 20-week exercise program consisting of 30 to 50 minutes of cycling once a week (30).

In another study, Hindawi and Amjaliyeh (2016) observed a significant decrease in CRP levels after 11 weeks of endurance training in sedentary middle-aged men (31). Some researchers believe that exercise programs combined with weight loss and body fat percentage as a major source of inflammatory cytokines such as interleukin-1 alpha and interferon alpha, are effective in reducing CRP (31). Training period time is also an important factor in CRP changes due to exercise, so most studies that have reported a decrease in CRP have used training programs with at least eight weeks (32). Numerous other factors may be involved in reducing CRP due to physical activity, including the characteristics of the subject (sex, basal fat cell, genetic background, early levels of CRP) (33). Decreased CRP levels it may be suggested that the reduction in inflammatory status is an important factor in improving insulin sensitivity (32). Previous studies have shown that CRP can be a prognosis, and diagnostic markers for cardiovascular disease and diabetes have been established as appropriate reference domains for CRP to identify disease severity and disease risk classification. 

However, prior to acceptance as a useful biomarker in the clinic for diseases, CRP measurement can support treatment as a marker and provide any diagnostic and prognostic information and can be used for medical and clinical measurements are used in the usual clinic and aerobic exercise can be useful for clinical decision making and improving the condition of type 1 diabetic patients.

One of the limitations of this study was to satisfy the patients and their families to participate in this study. Little is known about pathology, the role of CRP in type 1 diabetes, CAD, hypertension and cardiovascular diabetes. Further intervention studies such as the AT1 receptor antagonist should be performed to accept CRP as a predictor of diabetes and cardiovascular disease. A better understanding of CRP signaling pathways may help discover therapies for diabetes and cardiovascular complications.

## Funding:

Not grant

## Conflict of Interests:

All the authors decline that they have no conflict of interests
